# Supplementary material for: What Are the Key Anatomical Features for the Success of Nose-to-Brain Delivery? A Study of Powder Deposition in 3D-Printed Nasal Casts
Source: Pharmaceutics. 2023 Nov 23;15(12):2661. doi: 10.3390/pharmaceutics15122661 (PMC10747338; doi:10.3390/pharmaceutics15122661)
Supplement: Supplementary file 1 [file pharmaceutics-15-02661-s001.zip › pharmaceutics-2707119-supplementary.pdf]

S1: Details of the statistical method

Multivariate statistics is an ensemble of methods seeking to describe datasets whose outputs are constituted by more than one variable. In our case, the output variable is the overall deposition in the cast and the influence of each parameter. These variables are described by the coefficients of the statistical models extracted from the experiments. The goal of multivariate statistics is to group the variables that are linked by a shared relationship.
The most frequent of those statistics techniques is the principal component analysis (PCA). The principle of this method is to explain as much variability as reasonably achievable by using the least possible amount of variables. For that, the initial output variables are combined by linear transformation into a new set of output variables, the so-called principal components. The advantage of these new variables is that a given variable explains more variability than the following one. Consequently, it is possible to explain most of the variance (usually more than 70%) using a limited number of variables (usually 2 or 3) instead of the initial set.
The number of components to retain in the final analysis can be determined by multiple methods. The easiest one is setting the minimal variance explained by the x first components. Another technique is to use a scree plot, i.e. the representation of the variance explained by each component and looking for the elbow in the graph. Indeed, the elbow means that the benefit of adding a new component in term sof explanation is lower than the cost of increased complexity. Once the components that should be kept are known, the individuals can be represented in 2D spaces, called factorial planes, using two principal components as axes instead of the initial set of variables. Since the method aims to explain the maximal amount of variance by the first components, the points in the factorial planes are as spread as possible.
Another method, similar to principal component analysis, is exploratory factor analysis (EFA). They both use a similar approach of linear transformation to group a large number of variables into a smaller set of outputs based on the similarities between them. The difference between the two methods comes from the assumption made in EFA that there exists one or more latent variables that are not directly observed but that explain the results. The goal is thus to group the observed variables by the latent variables underlying them instead of explaining the most variance possible.
After this initial dimensionality-reduction step, other techniques can be applied to cluster the individuals in a certain number of groups sharing common characteristics. The two most used clustering methods are hierarchical clustring and centroid-based clustering, also called k-means clustering. Both techniques rely on the relative positions of the individuals in the space, either the observed output space or the transformed factorial plane.
Hierarchical clustering begins by grouping the two closest individuals and representing the resulting cluster by the median position of the initial points. The process is then reiterated by grouping the next-closest points, including those created by aggregation, until all the individuals are linked together in a single cluster.
The initial step of k-means clustering is determining the final number of clusters. This number can be determined by a priori knowledge, observation of the dataset or other techniques, such as using a previous hierarchical clustering, that does not need the number of clusters to be determined initially. The seeds, i.e. the initial center of the groups, are then placed in the space, either randomly or deterministically, and each individual is assigned to the cluster whose center is the closest. Once all individuals have been linked to a group, the

centers of these clusters are computed and serve as seeds for the next iteration. These steps are repeated until two subsequent iterations give the same clascsification.

S2: Coronal slices of the anatomies

Coronal slice of each anatomy used in the study. Note that C1 is slice of the STL file and C2 has been artificially created from C5 by filling the septum perforation.

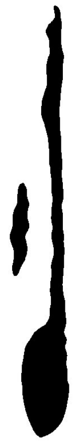	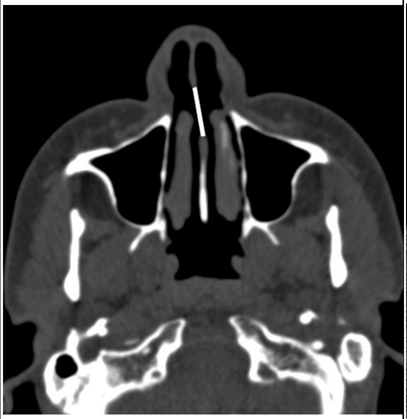	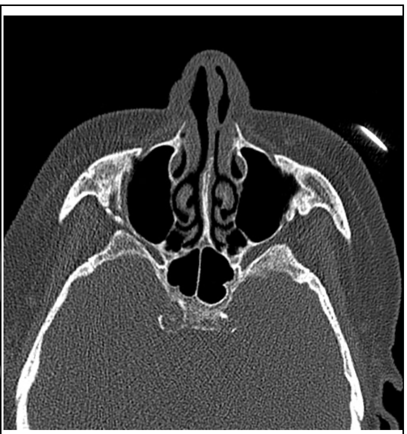
C1 – Standard	C2 – Normal	C3 – Normal
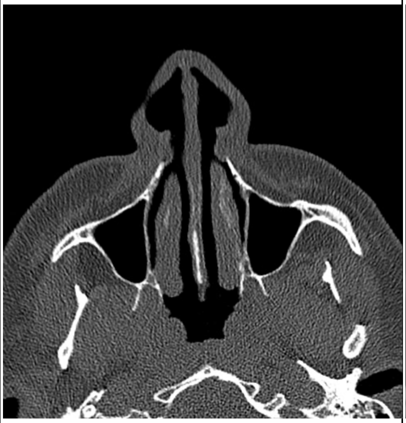	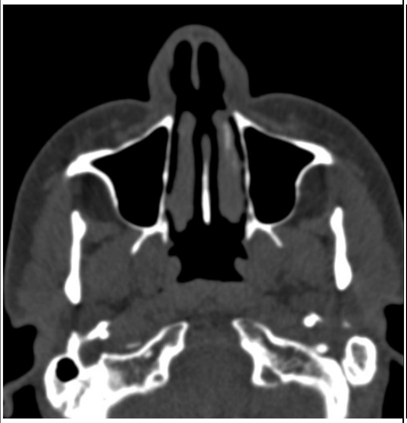	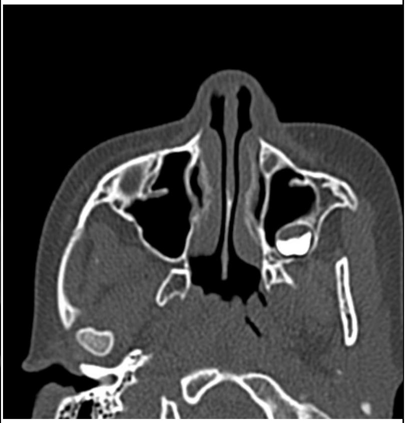
C4 – Normal	C5 – Perforation	C6 – Pediatric
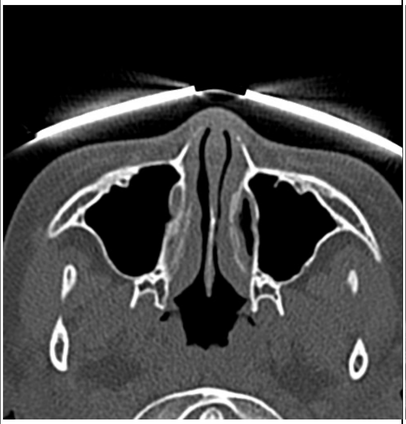	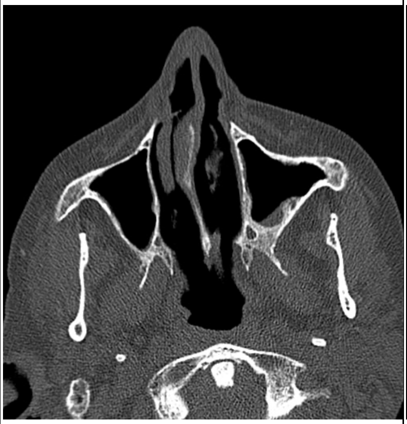	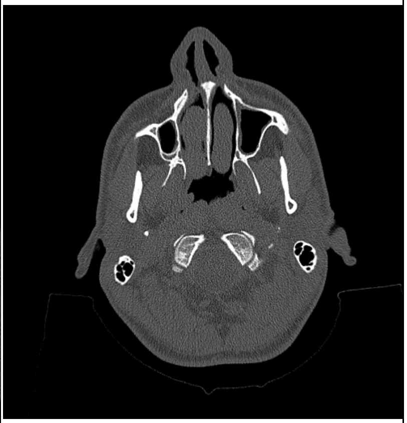
C7 – Pediatric	C8 – Septum deviation	C9 – Septum deviation

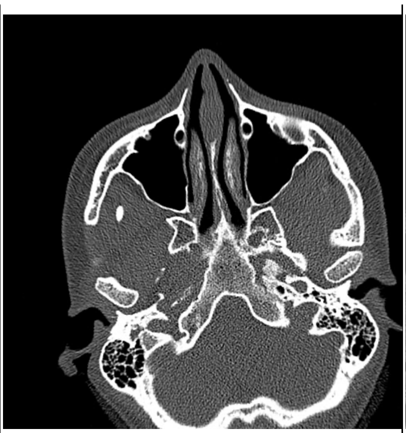	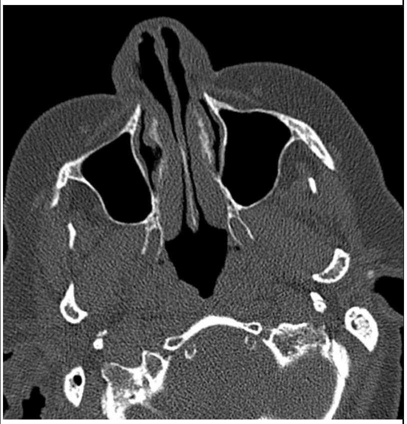	
C10 – Turbinate hypertrophy	C11 – Turbinate hypertrophy	

S3: Raw experimental results

C1	Factor 1	Factor 2	Factor 3	Response 1	Response 2	Response 3	Response 4	Response 5	Response 6
**Run**	Device	Angle (°)	Inspiratory flow (L/min)	Nostril	Olfactory region	Middle turbinate	Lower turbinates	Naso-pharynx	Post-nasal fraction
				%	%	%	%	%	%
**1**	Trivair	Center	0	18.6	1.2	30.0	38.2	8.8	3.1
**2**	Trivair	Center	0	20.9	3.3	33.5	35.0	5.5	1.8
**3**	Trivair	Direct	0	30.9	5.5	37.7	22.3	2.3	1.2
**4**	MIAT	Center	60	5.2	5.8	35.8	24.6	15.1	13.6
**5**	MIAT	Direct	0	29.6	6.1	28.9	28.8	3.6	3.0
**6**	MIAT	Direct	60	11.8	6.8	32.6	22.6	13.6	12.6
**7**	MIAT	Direct	15	11.4	8.2	32.0	20.6	15.3	12.6
**8**	MIAT	Center	0	9.7	9.1	43.4	17.0	15.5	5.2
**9**	MIAT	Direct	0	29.2	9.2	24.7	30.2	5.1	1.7
**10**	MIAT	Center	15	5.5	9.6	34.2	23.1	18.0	9.6
**11**	UDS	Center	60	6.9	10.5	36.0	24.5	15.5	6.6
**12**	MIAT	Center	15	10.0	12.0	30.2	21.8	15.8	10.2
**13**	MIAT	Center	0	18.2	14.6	32.3	26.9	5.1	3.0
**14**	UDS	Center	60	5.0	22.7	38.4	15.3	13.6	5.0
**15**	UDS	Direct	0	13.1	27.2	35.3	21.4	2.8	0.2
**16**	UDS	Center	15	7.1	29.5	27.4	16.8	15.3	3.9
**17**	UDS	Direct	0	14.0	31.2	38.3	13.8	2.5	0.2
**18**	UDS	Center	0	10.0	35.5	40.5	12.7	1.3	0.0

C2	Factor 1	Factor 2	Factor 3	Factor 4	Response 1	Response 2	Response 3	Response 4	Response 5	Response 6
**Run**	Device	Angle (°)	Inspiratory flow (L/min)	Side	Nostril	Olfactory region	Middle turbinate	Lower turbinates	Naso-pharynx	Post-nasal fraction
					%	%	%	%	%	%
**1**	MIAT	Center	60	Right	2.5	0.8	27.2	18.0	42.4	9.0
**2**	Trivair	Direct	0	Right	7.8	1.1	50.1	39.6	0.8	0.5
**3**	UDS	Center	15	Left	11.0	3.6	63.2	19.7	1.5	0.9
**4**	MIAT	Direct	60	Right	7.9	5.4	64.9	3.4	14.2	4.2
**5**	UDS	Center	60	Left	16.9	5.9	62.0	7.7	7.1	0.3
**6**	Trivair	Center	0	Left	7.0	6.7	50.3	18.4	16.2	1.4
**7**	MIAT	Direct	15	Left	6.8	7.1	65.7	11.5	6.8	2.1
**8**	MIAT	Center	60	Left	6.5	7.8	71.3	11.1	2.6	0.8
**9**	Trivair	Direct	0	Right	7.2	8.1	52.9	15.3	8.7	7.8
**10**	UDS	Center	0	Left	9.3	8.1	61.1	20.1	0.7	0.6
**11**	Trivair	Direct	0	Right	7.4	8.2	57.0	27.2	0.2	0.1
**12**	Trivair	Direct	0	Left	22.0	9.8	64.5	3.4	0.1	0.1
**13**	UDS	Center	0	Right	6.2	12.8	70.2	8.5	0.4	1.8
**14**	MIAT	Direct	60	Left	27.6	14.3	36.3	14.0	6.4	1.3
**15**	MIAT	Center	0	Right	7.2	16.2	49.2	17.0	7.4	3.0
**16**	MIAT	Center	0	Left	6.9	16.6	55.8	14.7	8.1	0.7
**17**	MIAT	Center	0	Left	6.9	16.6	55.8	14.7	8.1	0.7
**18**	UDS	Direct	15	Right	1.7	19.6	59.5	9.1	8.3	1.7
**19**	UDS	Direct	60	Left	8.7	22.9	64.2	1.0	2.9	0.3
**20**	UDS	Direct	60	Right	2.7	23.7	57.4	7.3	8.6	0.3
**21**	UDS	Direct	15	Left	11.5	27.7	56.0	3.5	0.5	0.7
**22**	MIAT	Center	15	Right	5.2	28.0	20.7	24.7	13.2	8.2
**23**	UDS	Direct	60	Right	2.8	32.6	54.6	3.6	5.9	0.4
**24**	MIAT	Direct	0	Right	10.2	33.6	31.9	8.0	8.5	7.7
**25**	UDS	Direct	0	Right	1.9	38.6	53.5	4.5	1.0	0.5
**26**	UDS	Direct	0	Right	1.9	38.6	53.5	4.5	1.0	0.5
**27**	UDS	Direct	15	Left	8.0	40.4	46.1	2.0	2.0	1.5

C3	Factor 1	Factor 2	Factor 3	Factor 4	Response 1	Response 2	Response 3	Response 4	Response 5	Response 6
**Run**	Device	Angle (°)	Inspiratory flow (L/min)	Side	Nostril	Olfactory region	Middle turbinate	Lower turbinates	Naso-pharynx	Post-nasal fraction
					%	%	%	%	%	%
**1**	TriVair	Direct	0	Right	67.2	1.7	22.2	5.1	1.8	2.0
**2**	TriVair	Direct	0	Left	49.4	2.1	28.4	13.5	5.3	1.2
**3**	UDS	Direct	15	Left	58.7	2.2	14.9	14.8	5.9	3.4
**4**	TriVair	Center	0	Left	71.3	3.0	13.9	10.1	0.9	0.9
**5**	MIAT	Direct	60	Left	63.9	3.7	29.0	1.4	0.6	1.3
**6**	MIAT	Center	15	Left	60.6	4.2	14.8	13.6	2.7	4.0
**7**	TriVair	Direct	0	Left	55.3	4.3	25.0	9.3	4.6	1.6
**8**	MIAT	Direct	15	Left	55.7	4.8	23.6	9.8	2.9	3.1
**9**	TriVair	Direct	0	Right	39.0	5.3	48.4	3.4	2.5	1.4
**10**	MIAT	Direct	15	Right	41.9	6.0	36.9	8.9	5.1	1.7
**11**	UDS	Direct	60	Right	26.8	10.1	47.0	4.5	11.0	0.5
**12**	UDS	Center	0	Left	46.0	10.2	33.1	9.5	0.7	0.5
**13**	UDS	Center	0	Left	47.7	11.5	32.6	7.6	0.6	0.2
**14**	MIAT	Center	60	Left	33.0	13.3	32.3	15.9	3.7	1.8
**15**	MIAT	Center	0	Right	45.8	15.4	33.3	3.8	1.2	0.5
**16**	UDS	Center	60	Left	38.6	16.4	35.9	4.8	3.5	0.7
**17**	TriVair	Center	0	Right	56.3	20.4	20.3	1.4	0.9	0.7
**18**	UDS	Center	0	Right	36.6	41.6	20.8	0.5	0.4	0.1
**19**	UDS	Center	15	Right	35.3	43.7	19.0	0.4	1.0	0.5

C5	Factor 1	Factor 2	Factor 3	Factor 4	Response 1	Response 2	Response 3	Response 4	Response 5	Response 6
**Run**	Device	Angle (°)	Inspiratory flow (L/min)	Side	Nostril	Olfactory region	Middle turbinate	Lower turbinates	Naso-pharynx	Post-nasal fraction
					%	%	%	%	%	%
**1**	Trivair	Center	0	Left	12.9	0.3	16.1	70.4	0.1	0.1
**2**	UDS	Center	15	Left	11.2	0.4	80.3	6.9	0.6	0.6
**3**	MIAT	Center	15	Left	1.0	0.5	62.3	20.2	10.7	5.2
**4**	UDS	Direct	15	Left	19.4	0.6	74.4	4.3	0.7	0.7
**5**	UDS	Direct	15	Right	19.4	0.6	74.4	4.3	0.7	0.7
**6**	MIAT	Direct	15	Left	6.9	1.0	75.4	12.4	2.7	1.6
**7**	Trivair	Center	0	Right	7.0	2.2	39.9	49.5	1.3	0.1
**8**	UDS	Center	0	Left	11.2	5.5	70.9	10.3	1.4	0.7
**9**	MIAT	Direct	60	Left	30.5	6.7	55.8	5.3	0.6	1.2
**10**	MIAT	Center	0	Left	39.8	8.5	28.1	20.3	1.7	1.6
**11**	MIAT	Center	15	Right	1.4	8.7	53.6	17.9	1.0	17.4
**12**	MIAT	Direct	0	Left	18.2	9.4	61.0	9.4	1.7	0.3
**13**	Trivair	Direct	0	Left	31.0	10.9	44.5	11.9	1.3	0.4
**14**	MIAT	Direct	15	Left	9.8	11.0	50.6	16.9	7.3	4.4
**15**	UDS	Center	60	Right	0.6	12.4	59.0	15.5	11.2	1.3
**16**	UDS	Center	60	Left	9.0	14.5	69.7	2.4	3.9	0.6
**17**	Trivair	Direct	0	Right	8.9	14.5	60.8	14.8	0.7	0.3
**18**	UDS	Direct	0	Left	6.9	14.8	73.0	4.9	0.3	0.1
**19**	UDS	Center	0	Left	7.2	17.2	65.5	9.1	0.7	0.2
**20**	MIAT	Center	60	Right	6.1	19.1	28.3	17.1	19.2	10.1
**21**	MIAT	Direct	15	Right	4.0	21.6	45.9	0.9	14.0	13.5
**22**	UDS	Direct	60	Left	15.7	22.0	59.1	1.6	1.4	0.2
**23**	UDS	Center	0	Right	2.7	24.2	60.8	10.0	1.9	0.5
**24**	MIAT	Direct	0	Right	14.6	25.5	30.2	6.6	18.3	4.8
**25**	UDS	Direct	15	Left	8.0	29.0	56.1	5.0	1.1	0.9
**26**	MIAT	Center	0	Right	1.9	31.2	47.1	12.3	6.5	0.9
**27**	UDS	Direct	60	Right	2.2	37.6	54.3	1.1	3.8	1.0
**28**	UDS	Center	15	Right	4.0	46.5	43.1	1.9	3.1	1.4
**29**	UDS	Direct	15	Right	1.8	47.7	41.4	2.9	4.2	1.9
**30**	UDS	Direct	0	Right	2.1	54.0	36.3	5.1	2.0	0.6

C6	Factor 1	Factor 2	Factor 3	Factor 4	Response 1	Response 2	Response 3	Response 4	Response 5	Response 6
**Run**	Device	Angle (°)	Inspiratory flow (L/min)	Side	Nostril	Olfactory region	Middle turbinate	Lower turbinates	Naso-pharynx	Post-nasal fraction
					%	%	%	%	%	%
**1**	MIAT	Center	60	Left	15.1	9.9	16.2	4.2	20.9	33.7
**2**	MIAT	Direct	60	Right	38.8	10.9	22.1	21.2	6.0	1.1
**3**	Trivair	Center	0	Left	27.2	12.3	18.8	24.2	8.8	8.8
**4**	MIAT	Center	15	Left	38.0	12.3	19.6	12.3	9.0	8.7
**5**	MIAT	Center	15	Right	25.2	20.7	26.0	11.9	12.4	3.8
**6**	MIAT	Center	60	Right	28.4	21.0	18.0	13.7	11.1	7.7
**7**	MIAT	Center	0	Right	21.5	22.9	27.9	17.3	6.7	3.7
**8**	MIAT	Direct	0	Right	25.4	27.3	23.4	13.0	8.2	2.7
**9**	Trivair	Direct	0	Right	13.4	28.6	27.9	15.7	13.0	1.4
**10**	MIAT	Direct	0	Right	23.8	31.8	30.5	6.1	7.2	0.6
**11**	Trivair	Direct	0	Right	14.9	32.3	26.8	15.3	9.6	1.1
**12**	UDS	Direct	60	Left	11.4	35.0	19.9	7.6	15.3	10.8
**13**	UDS	Center	0	Right	17.8	36.8	26.1	18.8	0.5	0.0
**14**	UDS	Direct	60	Left	14.7	49.9	20.4	5.7	6.5	2.8
**15**	UDS	Direct	15	Right	10.5	56.3	21.8	4.1	5.5	1.8
**16**	UDS	Direct	15	Right	15.1	59.1	14.6	5.4	4.6	1.3

C7	Factor 1	Factor 2	Factor 3	Factor 4	Response 1	Response 2	Response 3	Response 4	Response 5	Response 6
**Run**	Device	Angle (°)	Inspiratory flow (L/min)	Side	Nostril	Olfactory region	Middle turbinate	Lower turbinates	Naso-pharynx	Post-nasal fraction
					%	%	%	%	%	%
**1**	UDS	Center	60	Right	13.2	3.3	60.0	2.1	19.2	2.0
**2**	TriVair	Center	0	Left	45.5	1.0	42.2	5.5	4.3	1.5
**3**	TriVair	Center	0	Right	28.7	0.9	53.4	7.5	7.9	1.7
**4**	MIAT	Center	60	Left	49.9	7.3	30.7	1.5	6.7	3.8
**5**	UDS	Center	0	Right	23.2	8.0	57.2	6.3	4.7	0.5
**6**	TriVair	Direct	0	Left	46.8	5.1	42.0	2.3	2.2	1.6
**7**	MIAT	Direct	0	Right	49.8	2.2	33.2	10.1	3.4	1.3
**8**	MIAT	Direct	0	Left	52.8	1.5	26.9	5.0	11.7	2.1
**9**	UDS	Direct	15	Left	45.7	1.2	45.7	5.2	1.5	0.8
**10**	MIAT	Direct	60	Right	79.4	0.2	4.4	12.4	2.3	1.2
**11**	UDS	Center	60	Left	53.4	0.6	39.4	2.7	3.5	0.4
**12**	UDS	Center	15	Left	28.1	8.1	55.1	1.2	5.7	1.8
**13**	TriVair	Direct	0	Right	30.8	4.8	47.6	9.2	6.0	2.1
**14**	UDS	Direct	15	Right	31.9	0.3	51.9	2.1	12.1	1.7
**15**	UDS	Direct	0	Left	31.2	5.9	56.9	3.9	2.0	0.2
**16**	MIAT	Direct	15	Left	68.6	2.0	23.7	1.0	2.1	2.7
**17**	MIAT	Center	60	Right	35.8	6.8	41.9	4.6	6.2	4.7
**18**	UDS	Direct	60	Left	39.0	5.9	46.8	1.1	5.9	1.3
**19**	UDS	Direct	60	Right	29.3	5.2	47.6	9.6	7.3	1.1
**20**	MIAT	Direct	60	Left	29.9	2.9	42.8	2.8	13.8	7.9
**21**	MIAT	Center	0	Left	44.8	7.0	40.9	3.6	2.9	0.7
**22**	MIAT	Center	0	Right	33.2	11.0	42.2	9.5	3.9	0.3
**23**	UDS	Center	0	Left	27.1	12.3	57.9	1.2	1.3	0.2
**24**	TriVair	Direct	0	Left	53.4	6.1	37.4	0.8	2.3	0.0

C8	Factor 1	Factor 2	Factor 3	Factor 4	Response 1	Response 2	Response 3	Response 4	Response 5	Response 6
**Run**	Device	Angle (°)	Inspiratory flow (L/min)	Side	Nostril	Olfactory region	Middle turbinate	Lower turbinates	Naso-pharynx	Post-nasal fraction
					%	%	%	%	%	%
**1**	Trivair	Center	0	Right	24.1	3.5	37.5	30.7	3.8	0.3
**2**	Trivair	Direct	0	Left	34.8	12.6	16.3	34.5	1.5	0.3
**3**	UDS	Direct	60	Left	33.2	34.8	15.4	13.8	1.6	1.2
**4**	UDS	Center	0	Right	21.5	23.4	31.7	13.1	0.5	0.1
**5**	MIAT	Direct	15	Right	29.8	6.0	36.7	23.2	1.7	2.7
**6**	UDS	Direct	60	Left	24.5	18.8	22.2	21.5	1.9	1.7
**7**	MIAT	Direct	15	Right	29.5	2.9	36.7	24.7	3.5	2.8
**8**	MIAT	Center	0	Left	21.1	5.2	26.9	43.4	2.9	0.4
**9**	MIAT	Center	0	Left	12.9	4.2	37.3	44.4	0.7	0.4
**10**	Trivair	Direct	0	Left	34.9	17.7	17.6	26.3	1.9	1.6
**11**	Trivair	Center	0	Right	26.8	5.4	32.0	26.7	8.5	0.7
**12**	UDS	Center	15	Left	14.0	10.7	18.9	37.1	0.6	1.9
**13**	UDS	Direct	15	Left	25.6	14.2	22.6	28.5	0.6	1.6
**14**	MIAT	Direct	0	Left	37.0	0.3	1.7	51.3	9.4	0.3
**15**	MIAT	Direct	60	Left	34.1	13.0	19.5	30.3	1.1	1.9
**16**	MIAT	Center	60	Left	35.1	1.2	9.4	47.5	4.8	2.0
**17**	UDS	Direct	0	Right	20.9	5.4	60.1	12.9	0.5	0.1
**18**	UDS	Direct	60	Left	33.8	23.7	20.5	21.2	0.4	0.4

C9	Factor 1	Factor 2	Factor 3	Factor 4	Response 1	Response 2	Response 3	Response 4	Response 5	Response 6
Run	Device	Angle (°)	Inspiratory flow (L/min)	Side	Nostril	Olfactory region	Middle turbinate	Lower turbinates	Naso-pharynx	Post-nasal fraction
					%	%	%	%	%	%
1	UDS	Direct	0	Right	53.5	0.9	24.9	20.5	0.1	0.0
2	MIAT	Center	0	Right	57.3	1.8	21.8	18.8	0.2	0.1
3	Trivair	Center	0	Right	18.6	2.9	48.0	30.0	0.3	0.2
4	UDS	Center	15	Right	28.3	3.8	42.8	24.3	0.5	0.3
5	Trivair	Center	0	Right	17.0	3.8	43.1	35.7	0.4	0.0
6	MIAT	Direct	15	Right	20.3	4.0	55.2	19.0	1.3	0.1
7	UDS	Center	60	Right	25.0	6.8	50.4	16.2	1.1	0.5
8	Trivair	Direct	0	Left	31.1	6.9	51.8	9.5	0.6	0.0
9	UDS	Direct	0	Right	35.9	7.2	36.2	20.6	0.1	0.0
10	Trivair	Center	0	Right	11.1	7.4	61.0	20.1	0.3	0.1
11	Trivair	Direct	0	Right	22.3	13.0	55.2	9.2	0.2	0.2
12	Trivair	Center	0	Left	12.6	15.6	63.9	7.1	0.4	0.4
13	MIAT	Center	15	Left	37.6	18.6	40.6	2.6	0.3	0.2
14	Trivair	Direct	0	Left	20.0	24.0	50.4	4.9	0.1	0.5
15	UDS	Direct	15	Left	19.2	36.1	40.8	3.5	0.1	0.3
16	UDS	Center	0	Left	23.2	42.8	31.3	2.4	0.2	0.1

C10	Factor 1	Factor 2	Factor 3	Factor 4	Response 1	Response 2	Response 3	Response 4	Response 5	Response 6
Run	Device	Angle (°)	Inspiratory flow (L/min)	Side	Nostril	Olfactory region	Middle turbinate	Lower turbinates	Naso-pharynx	Post-nasal fraction
					%	%	%	%	%	%
1	TriVair	Center	0	Left	24.3	2.7	54.1	16.0	1.7	1.1
2	TriVair	Center	0	Right	52.5	0.6	33.4	12.6	0.7	0.2
3	UDS	Center	15	Right	24.2	4.1	57.3	10.8	2.1	1.6
4	TriVair	Direct	0	Left	51.7	0.5	32.2	14.7	0.6	0.3
5	TriVair	Center	0	Left	20.9	4.7	62.2	10.8	0.7	0.8
6	TriVair	Direct	0	Right	25.6	0.3	33.8	37.3	1.9	1.2
7	UDS	Direct	0	Right	28.3	3.2	53.3	13.6	1.3	0.3
8	MIAT	Center	60	Left	24.7	0.7	53.2	19.0	1.4	1.0
9	UDS	Direct	0	Left	25.7	4.0	55.8	13.1	1.2	0.2
10	MIAT	Direct	15	Right	37.6	1.2	22.9	36.1	1.4	0.9
11	TriVair	Direct	0	Left	54.8	1.0	26.2	16.3	0.9	0.8
12	UDS	Direct	60	Left	25.5	8.3	51.8	10.7	2.2	1.4
13	MIAT	Center	60	Right	20.9	12.5	34.6	21.1	6.0	4.9
14	MIAT	Center	0	Left	31.1	0.2	24.4	39.6	3.7	1.0

C11	Factor 1	Factor 2	Factor 3	Factor 4	Response 1	Response 2	Response 3	Response 4	Response 5	Response 6
**Run**	Device	Angle (°)	Inspiratory flow (L/min)	Side	Nostril	Olfactory region	Middle turbinate	Lower turbinates	Naso-pharynx	Post-nasal fraction
					%	%	%	%	%	%
**1**	MIAT	Direct	0	Left	77.5	0.0	3.1	19.4	0.0	0.0
**2**	UDS	Direct	60	Right	17.2	0.2	48.3	26.2	7.6	0.5
**3**	UDS	Center	0	Right	34.9	0.4	6.6	57.2	0.8	0.2
**4**	UDS	Center	60	Right	11.5	0.8	58.3	14.1	13.4	2.0
**5**	UDS	Center	60	Right	11.0	1.0	56.4	11.6	17.8	2.2
**6**	UDS	Center	60	Left	6.4	1.1	71.5	5.7	13.9	1.4
**7**	UDS	Direct	15	Left	19.7	1.1	54.6	12.0	11.6	1.0
**8**	Trivair	Center	0	Right	9.2	1.2	79.4	10.2	0.1	0.0
**9**	Trivair	Direct	0	Right	11.7	1.2	59.5	27.3	0.1	0.2
**10**	MIAT	Center	60	Right	18.6	2.7	61.6	10.9	5.6	0.6
**11**	MIAT	Direct	15	Left	14.5	3.2	46.9	35.1	0.1	0.2
**12**	MIAT	Direct	15	Right	23.5	4.8	62.1	6.7	1.4	1.6
**13**	Trivair	Center	0	Left	21.8	6.1	52.6	16.8	1.7	1.0
**14**	Trivair	Direct	0	Left	12.4	10.8	58.8	15.6	3.1	0.2
**15**	Trivair	Direct	0	Left	13.8	14.7	65.3	5.7	0.3	0.1
